# 4-(1-Adamantylmeth­yl)-*N*-(2-chloro-9-isopropyl-9*H*-purin-6-yl)aniline

**DOI:** 10.1107/S1600536809023629

**Published:** 2009-06-24

**Authors:** Michal Rouchal, Marek Nečas, Robert Vícha

**Affiliations:** a Department of Chemistry, Faculty of Technology, Tomas Bata University in Zlin, Nám. T. G. Masaryka 275, Zlín, 762 72, Czech Republic; b Department of Chemistry, Faculty of Science, Masaryk University in Brno, Kamenice 5, Brno-Bohunice, 625 00, Czech Republic

## Abstract

The asymmetric unit of the title compound, C_25_H_30_ClN_5_, consists of two mol­ecules with slightly different geometrical parameters. The dihedral angles between the purine and benzene rings are 39.54 (5) and 23.69 (5)° in the two mol­ecules. The adamantane cages consist of three fused cyclo­hexane rings in classical chair conformations, with C—C—C angles in the range 108 (2)–111 (2)°. In the crystal, mol­ecules are linked into dimers *via* two N—H⋯N hydrogen bonds.

## Related literature

The title compound was prepared according to a modification of the procedure of Fiorini & Abel (1998 ▶). For the synthesis and/or biological activity of related compounds, see: Hardcastle *et al.* (2002 ▶); Villhauer *et al.*, (2003 ▶). For related structures, see: Trávníček & Zatloukal (2004 ▶); Trávníček & Popa (2007*a* ▶,*b* ▶); Rouchal *et al.* (2009*a* ▶,*b* ▶).
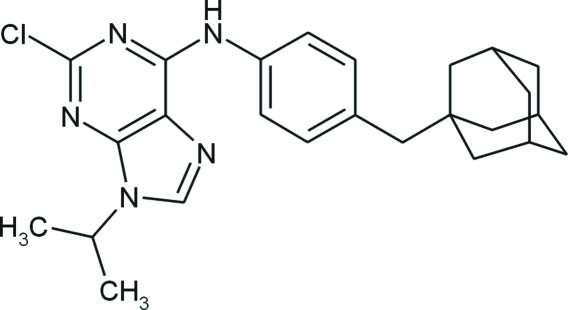


## Experimental

### 

#### Crystal data

C_25_H_30_ClN_5_
*M* *_r_* = 435.99
Triclinic, 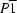

*a* = 11.731 (1) Å
*b* = 13.421 (1) Å
*c* = 15.531 (1) Å
α = 72.002 (7)°
β = 81.912 (7)°
γ = 79.688 (7)°
*V* = 2278.4 (3) Å^3^
*Z* = 4
Mo *K*α radiation
μ = 0.19 mm^−1^
*T* = 120 K
0.20 × 0.20 × 0.10 mm

#### Data collection

Kuma KM-4-CCD diffractometer
Absorption correction: multi-scan (**CrysAlis RED**; Oxford Diffraction, 2006 ▶) *T*_min_ = 0.962, *T*_max_ = 0.981
17173 measured reflections
7983 independent reflections
4502 reflections with *I* > 2σ(*I*)
*R*_int_ = 0.030

#### Refinement

*R*[*F* ^2^ > 2σ(*F* ^2^)] = 0.033
*wR*(*F* ^2^) = 0.081
*S* = 0.85
7983 reflections
563 parameters
H-atom parameters constrained
Δρ_max_ = 0.19 e Å^−3^
Δρ_min_ = −0.28 e Å^−3^

### 

Data collection: *CrysAlis CCD* (Oxford Diffraction, 2006 ▶); cell refinement: *CrysAlis CCD*; data reduction: *CrysAlis RED* (Oxford Diffraction, 2006 ▶); program(s) used to solve structure: *SHELXS97* (Sheldrick, 2008 ▶); program(s) used to refine structure: *SHELXL97* (Sheldrick, 2008 ▶); molecular graphics: *ORTEP-3* (Farrugia, 1997 ▶); software used to prepare material for publication: *SHELXL97*.

## Supplementary Material

Crystal structure: contains datablocks global, I. DOI: 10.1107/S1600536809023629/im2124sup1.cif

Structure factors: contains datablocks I. DOI: 10.1107/S1600536809023629/im2124Isup2.hkl

Additional supplementary materials:  crystallographic information; 3D view; checkCIF report

## Figures and Tables

**Table 1:** Hydrogen-bond geometry (Å, °)

*D*—H⋯*A*	*D*—H	H⋯*A*	*D*⋯*A*	*D*—H⋯*A*
N1—H1*A*⋯N54	0.88	2.14	2.940 (2)	152
N51—H51*A*⋯N4	0.88	2.27	3.026 (2)	144

**Fig. 1.**
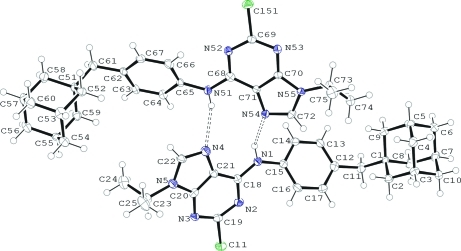
ORTEP plot of the asymmetric unit with atoms represented as 50% probability ellipsoids (H-bonds are denoted as dashed lines).

## supplementary crystallographic information

### Comment

The published structure represents a typical member of a new trisubstituted purine series currently synthesized in our laboratory. These molecules belong to the family of purine derivatives that exhibit a wide range of biological activities. Purine based molecules with suitable substituents at the most active centers can present low-molecular-weight inhibitors of cyclin-dependent kinases playing a crucial role in the regulation of the cell division cycle (Hardcastle *et al.*, 2002). A unique adamantane moiety is frequently linked into known medicaments or drug candidates with the aim to improve the biological properties of these structures, *e.g.* a novel potent hypoglycemic agent reported by Villhauer *et al.* (2003).

The title compound (Fig. 1) crystallizes with two geometrically slightly different molecules in the asymmetric unit that are linked into dimers by two N–H···N hydrogen bonds (Table 1). The dihedral angles between purine and benzene rings are 39.54 (5)° and 23.69 (5)°. The torsion angles describing the orientation of isopropyl, purine, benzene and adamantane moiety C22–N5–C23–H23A, C21–C18–N1–C15 and C17–C12–C11–C1 are -177.4 (2), 174.9 (2) and -94.6 (3)° respectively. The corresponding values of torsion angles for the second conformer are 144.0 (2), 173.9 (2) and -98.4 (2)° respectively.

### Experimental

The title compound was prepared according to a slightly modified literature procedure (Fiorini & Abel, 1998). 2,6-Dichloro-9-(propan-2-yl)-9*H*-purine (0.85 mmol, 196 mg) and 4-[(1-adamantyl)methyl]aniline hydrochloride (0.90 mmol, 250 mg) were dissolved in a mixture of DMF (2.5 ml) and triethylamine (1.70 mmol, 0.24 ml). The resulting solution was stirred at 363 K for required time (according to TLC). After the reaction was complete, the mixture was diluted with water and extracted with diethyl ether (5 times 15 ml). The connected organic layers were washed twice with brine and dried over sodium sulfate. The desired product was obtained by evaporation of the solvent in vacuum followed by purification of the crude product using column chromatography (silica gel; light petroleum/ethyl acetate (1:1 *v*/*v*) as a colorless crystalline powder (152 mg, 41%, mp 453–457 K). The single crystals suitable for X-ray analysis were grown by liquid diffusion (acetone/hexane, 1:3 *v*/*v*) at room temperature within 24 h.

### Refinement

Hydrogen atoms were positioned geometrically and refined as riding using standard *SHELXTL* facilities, with their *U*_iso_ set to either 1.2*U*_eq_ or 1.5*U*_eq_ (methyl) of their parent atoms.

### Crystal data

**Table:**

C_25_H_30_ClN_5_	*Z* = 4
*M**_r_* = 435.99	*F*(000) = 928
Triclinic, *P*1	*D*_x_ = 1.271 Mg m^−^^3^
Hall symbol: -P 1	Melting point: 455 K
*a* = 11.731 (1) Å	Mo *K*α radiation, λ = 0.71073 Å
*b* = 13.421 (1) Å	Cell parameters from 7983 reflections
*c* = 15.531 (1) Å	θ = 2.8–25.0°
α = 72.002 (7)°	µ = 0.19 mm^−^^1^
β = 81.912 (7)°	*T* = 120 K
γ = 79.688 (7)°	Plate, colourless
*V* = 2278.4 (3) Å^3^	0.20 × 0.20 × 0.10 mm

### Data collection

**Table:**

Kuma KM-4-CCD diffractometer	7983 independent reflections
Radiation source: fine-focus sealed tube	4502 reflections with *I* > 2σ(*I*)
graphite	*R*_int_ = 0.030
Detector resolution: 0.06 pixels mm^-1^	θ_max_ = 25.0°, θ_min_ = 2.8°
ω scans	*h* = −13→13
Absorption correction: multi-scan (*CrysAlis RED*; Oxford Diffraction, 2006)	*k* = −15→10
*T*_min_ = 0.962, *T*_max_ = 0.981	*l* = −18→18
17173 measured reflections	

### Refinement

**Table:**

Refinement on *F*^2^	Primary atom site location: structure-invariant direct methods
Least-squares matrix: full	Secondary atom site location: difference Fourier map
*R*[*F*^2^ > 2σ(*F*^2^)] = 0.033	Hydrogen site location: inferred from neighbouring sites
*wR*(*F*^2^) = 0.081	H-atom parameters constrained
*S* = 0.85	*w* = 1/[σ^2^(*F*_o_^2^) + (0.0366*P*)^2^] where *P* = (*F*_o_^2^ + 2*F*_c_^2^)/3
7983 reflections	(Δ/σ)_max_ = 0.002
563 parameters	Δρ_max_ = 0.19 e Å^−^^3^
0 restraints	Δρ_min_ = −0.28 e Å^−^^3^

### Special details

**Table:**

Geometry. All e.s.d.'s (except the e.s.d. in the dihedral angle between two l.s. planes) are estimated using the full covariance matrix. The cell e.s.d.'s are taken into account individually in the estimation of e.s.d.'s in distances, angles and torsion angles; correlations between e.s.d.'s in cell parameters are only used when they are defined by crystal symmetry. An approximate (isotropic) treatment of cell e.s.d.'s is used for estimating e.s.d.'s involving l.s. planes.
Refinement. Refinement of *F*^2^ against ALL reflections. The weighted *R*-factor *wR* and goodness of fit *S* are based on *F*^2^, conventional *R*-factors *R* are based on *F*, with *F* set to zero for negative *F*^2^. The threshold expression of *F*^2^ > 2σ(*F*^2^) is used only for calculating *R*-factors(gt) *etc*. and is not relevant to the choice of reflections for refinement. *R*-factors based on *F*^2^ are statistically about twice as large as those based on *F*, and *R*- factors based on ALL data will be even larger.

### Fractional atomic coordinates and isotropic or equivalent isotropic displacement parameters (Å^2^)

**Table:**

	*x*	*y*	*z*	*U*_iso_*/*U*_eq_	
Cl1	0.66480 (5)	0.02196 (4)	1.09703 (4)	0.02989 (16)	
N1	0.47703 (14)	0.31228 (13)	0.84935 (11)	0.0212 (4)	
H1A	0.4662	0.3352	0.7911	0.025*	
N2	0.56519 (14)	0.16913 (14)	0.96467 (11)	0.0213 (4)	
N3	0.64870 (14)	0.00011 (13)	0.93892 (11)	0.0203 (4)	
N4	0.54147 (15)	0.16914 (14)	0.72689 (11)	0.0253 (5)	
N5	0.62661 (14)	−0.00033 (14)	0.78420 (11)	0.0233 (4)	
C1	0.15565 (17)	0.67309 (16)	1.02665 (14)	0.0192 (5)	
C2	0.07127 (18)	0.59104 (16)	1.05850 (14)	0.0228 (5)	
H2B	0.0799	0.5510	1.1230	0.027*	
H2C	0.0905	0.5403	1.0222	0.027*	
C3	−0.05581 (18)	0.64537 (17)	1.04799 (14)	0.0230 (5)	
H3B	−0.1093	0.5906	1.0689	0.028*	
C4	−0.06925 (18)	0.70646 (17)	0.94756 (14)	0.0252 (6)	
H4B	−0.0509	0.6567	0.9103	0.030*	
H4C	−0.1507	0.7411	0.9404	0.030*	
C5	0.01297 (18)	0.79041 (17)	0.91466 (14)	0.0258 (6)	
H5A	0.0042	0.8296	0.8492	0.031*	
C6	−0.01802 (19)	0.86830 (16)	0.97223 (14)	0.0261 (6)	
H6A	0.0340	0.9233	0.9514	0.031*	
H6B	−0.0992	0.9037	0.9652	0.031*	
C7	−0.00410 (18)	0.80685 (17)	1.07278 (14)	0.0256 (6)	
H7A	−0.0234	0.8571	1.1104	0.031*	
C8	0.12254 (18)	0.75150 (17)	1.08348 (14)	0.0237 (5)	
H8A	0.1759	0.8054	1.0637	0.028*	
H8B	0.1314	0.7131	1.1483	0.028*	
C9	0.13970 (17)	0.73551 (17)	0.92593 (13)	0.0221 (5)	
H9A	0.1930	0.7894	0.9047	0.027*	
H9B	0.1599	0.6865	0.8881	0.027*	
C10	−0.08652 (18)	0.72313 (16)	1.10517 (14)	0.0239 (5)	
H10A	−0.1680	0.7579	1.0987	0.029*	
H10B	−0.0789	0.6845	1.1701	0.029*	
C11	0.28362 (18)	0.62051 (17)	1.03993 (14)	0.0247 (5)	
H11A	0.3334	0.6773	1.0202	0.030*	
H11B	0.2898	0.5866	1.1058	0.030*	
C12	0.33367 (17)	0.53798 (18)	0.99061 (14)	0.0216 (5)	
C13	0.38638 (17)	0.56703 (17)	0.90161 (14)	0.0222 (5)	
H13A	0.3888	0.6400	0.8713	0.027*	
C14	0.43523 (17)	0.49333 (17)	0.85604 (14)	0.0221 (5)	
H14A	0.4703	0.5159	0.7956	0.027*	
C15	0.43260 (17)	0.38575 (17)	0.89960 (14)	0.0186 (5)	
C16	0.38099 (18)	0.35447 (17)	0.98847 (14)	0.0237 (5)	
H16A	0.3788	0.2815	1.0188	0.028*	
C17	0.33275 (18)	0.42993 (17)	1.03266 (14)	0.0251 (6)	
H17A	0.2981	0.4073	1.0932	0.030*	
C18	0.53343 (17)	0.21243 (17)	0.87816 (14)	0.0183 (5)	
C19	0.62045 (18)	0.06941 (18)	0.98556 (14)	0.0210 (5)	
C20	0.61511 (18)	0.04658 (17)	0.85336 (14)	0.0205 (5)	
C21	0.56177 (17)	0.15023 (17)	0.81755 (14)	0.0198 (5)	
C22	0.58096 (18)	0.07720 (17)	0.71132 (14)	0.0250 (6)	
H22A	0.5781	0.0655	0.6544	0.030*	
C23	0.67589 (19)	−0.11099 (17)	0.78976 (15)	0.0260 (6)	
H23A	0.6980	−0.1473	0.8530	0.031*	
C24	0.7855 (2)	−0.11393 (19)	0.72457 (17)	0.0487 (8)	
H24A	0.8410	−0.0757	0.7383	0.073*	
H24B	0.7653	−0.0803	0.6620	0.073*	
H24C	0.8205	−0.1876	0.7315	0.073*	
C25	0.5850 (2)	−0.16786 (18)	0.77139 (17)	0.0418 (7)	
H25A	0.5161	−0.1638	0.8146	0.063*	
H25B	0.6172	−0.2423	0.7785	0.063*	
H25C	0.5631	−0.1342	0.7092	0.063*	
Cl51	0.28331 (5)	0.58057 (5)	0.30212 (4)	0.02837 (15)	
N51	0.52129 (14)	0.32715 (13)	0.54043 (11)	0.0190 (4)	
H51A	0.5363	0.3069	0.5977	0.023*	
N52	0.40140 (14)	0.44895 (13)	0.43181 (11)	0.0181 (4)	
N53	0.22379 (14)	0.55319 (13)	0.47526 (11)	0.0181 (4)	
N54	0.35856 (14)	0.41024 (13)	0.68229 (11)	0.0186 (4)	
N55	0.19479 (14)	0.52510 (13)	0.63989 (11)	0.0175 (4)	
C51	0.84156 (17)	−0.02070 (16)	0.35781 (13)	0.0178 (5)	
C52	0.72001 (17)	−0.04891 (16)	0.35767 (14)	0.0221 (5)	
H52B	0.6931	−0.0179	0.2961	0.027*	
H52C	0.6640	−0.0188	0.4003	0.027*	
C53	0.72440 (19)	−0.17002 (17)	0.38639 (15)	0.0266 (6)	
H53B	0.6452	−0.1873	0.3855	0.032*	
C54	0.76341 (19)	−0.21829 (17)	0.48350 (14)	0.0288 (6)	
H54B	0.7077	−0.1889	0.5267	0.035*	
H54C	0.7648	−0.2960	0.5025	0.035*	
C55	0.88519 (19)	−0.19224 (16)	0.48462 (14)	0.0242 (5)	
H55A	0.9108	−0.2227	0.5474	0.029*	
C56	0.97131 (19)	−0.23935 (17)	0.41818 (14)	0.0261 (6)	
H56A	1.0503	−0.2236	0.4192	0.031*	
H56B	0.9741	−0.3172	0.4368	0.031*	
C57	0.93224 (18)	−0.19142 (16)	0.32160 (14)	0.0241 (5)	
H57A	0.9883	−0.2218	0.2783	0.029*	
C58	0.92764 (18)	−0.06962 (16)	0.29220 (14)	0.0255 (6)	
H58A	1.0062	−0.0519	0.2917	0.031*	
H58B	0.9031	−0.0394	0.2298	0.031*	
C59	0.88055 (18)	−0.07101 (16)	0.45488 (14)	0.0227 (5)	
H59A	0.9585	−0.0534	0.4566	0.027*	
H59B	0.8255	−0.0410	0.4980	0.027*	
C60	0.81062 (19)	−0.21660 (17)	0.31982 (15)	0.0291 (6)	
H60A	0.8121	−0.2942	0.3373	0.035*	
H60B	0.7856	−0.1860	0.2576	0.035*	
C61	0.84161 (18)	0.10059 (15)	0.32633 (14)	0.0228 (5)	
H61A	0.9212	0.1143	0.3283	0.027*	
H61B	0.8234	0.1279	0.2621	0.027*	
C62	0.75770 (17)	0.16313 (15)	0.38065 (14)	0.0183 (5)	
C63	0.78513 (18)	0.17167 (15)	0.46234 (14)	0.0206 (5)	
H63A	0.8586	0.1388	0.4839	0.025*	
C64	0.70683 (17)	0.22741 (15)	0.51275 (14)	0.0195 (5)	
H64A	0.7273	0.2323	0.5682	0.023*	
C65	0.59856 (17)	0.27620 (15)	0.48277 (14)	0.0174 (5)	
C66	0.56996 (18)	0.27038 (16)	0.40016 (14)	0.0233 (5)	
H66A	0.4969	0.3039	0.3782	0.028*	
C67	0.65035 (19)	0.21459 (16)	0.35056 (14)	0.0234 (5)	
H67A	0.6311	0.2116	0.2941	0.028*	
C68	0.42732 (17)	0.40287 (16)	0.51939 (14)	0.0179 (5)	
C69	0.30520 (18)	0.52051 (16)	0.41747 (14)	0.0185 (5)	
C70	0.25453 (17)	0.50790 (15)	0.56185 (13)	0.0160 (5)	
C71	0.35421 (17)	0.43674 (15)	0.58869 (13)	0.0157 (5)	
C72	0.26190 (18)	0.46506 (16)	0.70871 (14)	0.0199 (5)	
H72A	0.2406	0.4631	0.7705	0.024*	
C73	0.08037 (17)	0.59161 (16)	0.64500 (14)	0.0192 (5)	
H73A	0.0789	0.6554	0.5905	0.023*	
C74	0.06466 (19)	0.62912 (17)	0.72953 (14)	0.0261 (6)	
H74A	0.1307	0.6644	0.7301	0.039*	
H74B	0.0607	0.5682	0.7839	0.039*	
H74C	−0.0075	0.6790	0.7290	0.039*	
C75	−0.01608 (18)	0.53080 (18)	0.64186 (15)	0.0304 (6)	
H75A	−0.0031	0.5097	0.5858	0.046*	
H75B	−0.0914	0.5761	0.6432	0.046*	
H75C	−0.0159	0.4676	0.6945	0.046*	

### Atomic displacement parameters (Å^2^)

**Table:**

	*U*^11^	*U*^22^	*U*^33^	*U*^12^	*U*^13^	*U*^23^
Cl1	0.0341 (4)	0.0329 (4)	0.0208 (3)	−0.0003 (3)	−0.0101 (3)	−0.0043 (3)
N1	0.0266 (11)	0.0203 (11)	0.0170 (10)	0.0020 (9)	−0.0042 (8)	−0.0079 (9)
N2	0.0215 (10)	0.0241 (12)	0.0188 (10)	−0.0026 (9)	−0.0046 (8)	−0.0064 (9)
N3	0.0234 (11)	0.0186 (11)	0.0179 (10)	0.0003 (8)	−0.0045 (8)	−0.0044 (9)
N4	0.0317 (11)	0.0236 (12)	0.0217 (11)	0.0007 (9)	−0.0068 (9)	−0.0088 (9)
N5	0.0262 (11)	0.0203 (11)	0.0227 (11)	0.0001 (9)	−0.0042 (8)	−0.0061 (9)
C1	0.0206 (12)	0.0184 (13)	0.0202 (12)	0.0007 (10)	−0.0006 (9)	−0.0105 (10)
C2	0.0313 (14)	0.0195 (13)	0.0170 (12)	−0.0022 (11)	−0.0026 (10)	−0.0049 (10)
C3	0.0229 (13)	0.0242 (14)	0.0213 (13)	−0.0050 (11)	−0.0002 (10)	−0.0056 (11)
C4	0.0210 (13)	0.0306 (15)	0.0244 (13)	−0.0016 (11)	−0.0030 (10)	−0.0096 (11)
C5	0.0269 (13)	0.0289 (14)	0.0168 (12)	−0.0010 (11)	−0.0040 (10)	−0.0005 (11)
C6	0.0266 (14)	0.0204 (14)	0.0263 (13)	0.0013 (11)	−0.0001 (10)	−0.0036 (11)
C7	0.0308 (14)	0.0257 (14)	0.0221 (13)	−0.0022 (11)	0.0027 (10)	−0.0128 (11)
C8	0.0282 (14)	0.0253 (14)	0.0195 (13)	−0.0032 (11)	−0.0015 (10)	−0.0096 (11)
C9	0.0222 (13)	0.0255 (14)	0.0184 (12)	−0.0059 (11)	0.0003 (10)	−0.0056 (10)
C10	0.0227 (13)	0.0244 (14)	0.0202 (13)	0.0045 (11)	−0.0006 (10)	−0.0052 (11)
C11	0.0244 (13)	0.0293 (14)	0.0239 (13)	−0.0016 (11)	−0.0056 (10)	−0.0127 (11)
C12	0.0149 (12)	0.0286 (15)	0.0235 (13)	−0.0011 (10)	−0.0070 (10)	−0.0096 (11)
C13	0.0220 (13)	0.0167 (13)	0.0253 (14)	0.0000 (10)	−0.0032 (10)	−0.0033 (11)
C14	0.0217 (13)	0.0233 (14)	0.0174 (12)	−0.0013 (11)	−0.0016 (10)	−0.0012 (11)
C15	0.0156 (12)	0.0224 (14)	0.0175 (12)	−0.0003 (10)	−0.0057 (9)	−0.0051 (11)
C16	0.0250 (13)	0.0174 (13)	0.0253 (14)	0.0007 (11)	−0.0049 (10)	−0.0020 (11)
C17	0.0271 (14)	0.0274 (15)	0.0173 (12)	0.0004 (11)	−0.0022 (10)	−0.0040 (11)
C18	0.0158 (12)	0.0171 (13)	0.0227 (13)	−0.0029 (10)	−0.0023 (10)	−0.0063 (11)
C19	0.0184 (12)	0.0255 (15)	0.0177 (12)	−0.0045 (11)	−0.0048 (10)	−0.0020 (11)
C20	0.0192 (12)	0.0255 (14)	0.0182 (13)	−0.0058 (10)	0.0002 (10)	−0.0077 (11)
C21	0.0197 (12)	0.0188 (13)	0.0215 (13)	−0.0012 (10)	−0.0058 (10)	−0.0061 (11)
C22	0.0356 (14)	0.0191 (14)	0.0184 (13)	0.0027 (11)	−0.0086 (11)	−0.0038 (11)
C23	0.0312 (14)	0.0193 (14)	0.0252 (13)	0.0056 (11)	−0.0042 (11)	−0.0074 (11)
C24	0.0436 (17)	0.0399 (18)	0.0519 (18)	0.0125 (14)	0.0044 (14)	−0.0121 (14)
C25	0.0486 (17)	0.0218 (15)	0.0589 (18)	−0.0019 (13)	−0.0133 (14)	−0.0153 (13)
Cl51	0.0277 (3)	0.0355 (4)	0.0161 (3)	0.0008 (3)	−0.0039 (2)	−0.0012 (3)
N51	0.0185 (10)	0.0229 (11)	0.0162 (10)	0.0018 (9)	−0.0042 (8)	−0.0082 (8)
N52	0.0168 (10)	0.0177 (11)	0.0201 (10)	−0.0023 (8)	−0.0016 (8)	−0.0060 (8)
N53	0.0177 (10)	0.0173 (10)	0.0193 (10)	−0.0014 (8)	−0.0012 (8)	−0.0062 (8)
N54	0.0199 (10)	0.0188 (11)	0.0164 (10)	0.0014 (8)	−0.0025 (8)	−0.0063 (8)
N55	0.0177 (10)	0.0186 (11)	0.0142 (10)	0.0006 (8)	−0.0036 (8)	−0.0027 (8)
C51	0.0188 (12)	0.0155 (13)	0.0193 (12)	−0.0001 (10)	0.0017 (9)	−0.0082 (10)
C52	0.0225 (13)	0.0222 (14)	0.0210 (12)	0.0010 (10)	−0.0059 (10)	−0.0061 (10)
C53	0.0234 (13)	0.0270 (15)	0.0327 (14)	−0.0081 (11)	−0.0049 (11)	−0.0098 (12)
C54	0.0354 (15)	0.0186 (14)	0.0304 (14)	−0.0040 (11)	0.0003 (11)	−0.0059 (11)
C55	0.0352 (14)	0.0189 (14)	0.0171 (12)	0.0014 (11)	−0.0118 (10)	−0.0020 (10)
C56	0.0284 (14)	0.0183 (13)	0.0331 (14)	0.0020 (11)	−0.0105 (11)	−0.0094 (11)
C57	0.0270 (13)	0.0208 (14)	0.0273 (13)	0.0018 (11)	0.0005 (10)	−0.0153 (11)
C58	0.0256 (13)	0.0259 (14)	0.0268 (13)	−0.0050 (11)	0.0024 (10)	−0.0116 (11)
C59	0.0251 (13)	0.0212 (14)	0.0247 (13)	−0.0019 (10)	−0.0037 (10)	−0.0110 (11)
C60	0.0370 (15)	0.0225 (14)	0.0325 (14)	−0.0040 (12)	−0.0104 (11)	−0.0118 (12)
C61	0.0233 (13)	0.0183 (13)	0.0250 (13)	−0.0029 (10)	0.0023 (10)	−0.0058 (11)
C62	0.0217 (12)	0.0105 (12)	0.0200 (12)	−0.0032 (10)	0.0038 (10)	−0.0025 (10)
C63	0.0194 (12)	0.0147 (13)	0.0272 (13)	−0.0001 (10)	−0.0017 (10)	−0.0069 (11)
C64	0.0183 (12)	0.0199 (13)	0.0218 (12)	−0.0027 (10)	−0.0042 (10)	−0.0074 (11)
C65	0.0178 (12)	0.0110 (12)	0.0224 (13)	−0.0033 (10)	0.0014 (10)	−0.0043 (10)
C66	0.0224 (13)	0.0227 (14)	0.0229 (13)	0.0030 (11)	−0.0083 (10)	−0.0047 (11)
C67	0.0334 (14)	0.0195 (13)	0.0160 (12)	0.0000 (11)	−0.0045 (10)	−0.0044 (10)
C68	0.0156 (12)	0.0160 (13)	0.0238 (13)	−0.0080 (10)	−0.0025 (10)	−0.0046 (10)
C69	0.0188 (12)	0.0191 (13)	0.0180 (12)	−0.0032 (10)	−0.0044 (10)	−0.0045 (10)
C70	0.0173 (12)	0.0150 (12)	0.0159 (12)	−0.0058 (10)	−0.0012 (9)	−0.0029 (10)
C71	0.0176 (12)	0.0138 (12)	0.0144 (12)	−0.0008 (10)	−0.0022 (9)	−0.0028 (10)
C72	0.0218 (13)	0.0247 (14)	0.0161 (12)	−0.0043 (11)	0.0001 (10)	−0.0103 (11)
C73	0.0169 (12)	0.0177 (13)	0.0204 (12)	0.0027 (10)	−0.0006 (9)	−0.0051 (10)
C74	0.0273 (13)	0.0223 (14)	0.0258 (13)	0.0020 (11)	0.0008 (10)	−0.0073 (11)
C75	0.0236 (13)	0.0323 (15)	0.0367 (15)	−0.0048 (11)	−0.0045 (11)	−0.0110 (12)

### Geometric parameters (Å, °)

**Table:**

Cl1—C19	1.768 (2)	Cl51—C69	1.757 (2)
N1—C18	1.352 (2)	N51—C68	1.360 (2)
N1—C15	1.425 (2)	N51—C65	1.421 (2)
N1—H1A	0.8800	N51—H51A	0.8800
N2—C19	1.340 (2)	N52—C69	1.340 (2)
N2—C18	1.365 (2)	N52—C68	1.362 (2)
N3—C19	1.318 (2)	N53—C69	1.327 (2)
N3—C20	1.363 (2)	N53—C70	1.364 (2)
N4—C22	1.321 (2)	N54—C72	1.324 (2)
N4—C21	1.398 (2)	N54—C71	1.392 (2)
N5—C22	1.376 (2)	N55—C72	1.374 (2)
N5—C20	1.384 (2)	N55—C70	1.375 (2)
N5—C23	1.475 (2)	N55—C73	1.480 (2)
C1—C2	1.534 (3)	C51—C52	1.540 (3)
C1—C8	1.539 (3)	C51—C58	1.541 (3)
C1—C9	1.549 (3)	C51—C59	1.547 (3)
C1—C11	1.553 (3)	C51—C61	1.548 (3)
C2—C3	1.545 (3)	C52—C53	1.540 (3)
C2—H2B	0.9900	C52—H52B	0.9900
C2—H2C	0.9900	C52—H52C	0.9900
C3—C10	1.535 (3)	C53—C60	1.537 (3)
C3—C4	1.537 (3)	C53—C54	1.545 (3)
C3—H3B	1.0000	C53—H53B	1.0000
C4—C5	1.535 (3)	C54—C55	1.534 (3)
C4—H4B	0.9900	C54—H54B	0.9900
C4—H4C	0.9900	C54—H54C	0.9900
C5—C6	1.541 (3)	C55—C56	1.538 (3)
C5—C9	1.544 (3)	C55—C59	1.541 (3)
C5—H5A	1.0000	C55—H55A	1.0000
C6—C7	1.541 (3)	C56—C57	1.538 (3)
C6—H6A	0.9900	C56—H56A	0.9900
C6—H6B	0.9900	C56—H56B	0.9900
C7—C10	1.534 (3)	C57—C60	1.530 (3)
C7—C8	1.544 (3)	C57—C58	1.548 (3)
C7—H7A	1.0000	C57—H57A	1.0000
C8—H8A	0.9900	C58—H58A	0.9900
C8—H8B	0.9900	C58—H58B	0.9900
C9—H9A	0.9900	C59—H59A	0.9900
C9—H9B	0.9900	C59—H59B	0.9900
C10—H10A	0.9900	C60—H60A	0.9900
C10—H10B	0.9900	C60—H60B	0.9900
C11—C12	1.524 (3)	C61—C62	1.514 (3)
C11—H11A	0.9900	C61—H61A	0.9900
C11—H11B	0.9900	C61—H61B	0.9900
C12—C17	1.396 (3)	C62—C67	1.391 (3)
C12—C13	1.399 (3)	C62—C63	1.394 (3)
C13—C14	1.387 (3)	C63—C64	1.389 (3)
C13—H13A	0.9500	C63—H63A	0.9500
C14—C15	1.396 (3)	C64—C65	1.392 (3)
C14—H14A	0.9500	C64—H64A	0.9500
C15—C16	1.395 (3)	C65—C66	1.399 (3)
C16—C17	1.390 (3)	C66—C67	1.394 (3)
C16—H16A	0.9500	C66—H66A	0.9500
C17—H17A	0.9500	C67—H67A	0.9500
C18—C21	1.411 (3)	C68—C71	1.415 (3)
C20—C21	1.394 (3)	C70—C71	1.394 (3)
C22—H22A	0.9500	C72—H72A	0.9500
C23—C25	1.521 (3)	C73—C74	1.523 (3)
C23—C24	1.523 (3)	C73—C75	1.523 (3)
C23—H23A	1.0000	C73—H73A	1.0000
C24—H24A	0.9800	C74—H74A	0.9800
C24—H24B	0.9800	C74—H74B	0.9800
C24—H24C	0.9800	C74—H74C	0.9800
C25—H25A	0.9800	C75—H75A	0.9800
C25—H25B	0.9800	C75—H75B	0.9800
C25—H25C	0.9800	C75—H75C	0.9800
			
C18—N1—C15	129.36 (18)	C68—N51—C65	129.02 (17)
C18—N1—H1A	115.3	C68—N51—H51A	115.5
C15—N1—H1A	115.3	C65—N51—H51A	115.5
C19—N2—C18	116.78 (18)	C69—N52—C68	117.47 (17)
C19—N3—C20	108.67 (17)	C69—N53—C70	109.58 (16)
C22—N4—C21	103.30 (17)	C72—N54—C71	102.93 (16)
C22—N5—C20	105.48 (17)	C72—N55—C70	105.18 (16)
C22—N5—C23	128.16 (17)	C72—N55—C73	129.36 (17)
C20—N5—C23	126.35 (17)	C70—N55—C73	125.42 (16)
C2—C1—C8	108.20 (17)	C52—C51—C58	108.83 (16)
C2—C1—C9	108.59 (16)	C52—C51—C59	108.02 (16)
C8—C1—C9	108.03 (17)	C58—C51—C59	108.37 (16)
C2—C1—C11	111.68 (17)	C52—C51—C61	111.60 (16)
C8—C1—C11	108.59 (16)	C58—C51—C61	108.54 (16)
C9—C1—C11	111.63 (17)	C59—C51—C61	111.40 (16)
C1—C2—C3	110.83 (17)	C51—C52—C53	110.02 (16)
C1—C2—H2B	109.5	C51—C52—H52B	109.7
C3—C2—H2B	109.5	C53—C52—H52B	109.7
C1—C2—H2C	109.5	C51—C52—H52C	109.7
C3—C2—H2C	109.5	C53—C52—H52C	109.7
H2B—C2—H2C	108.1	H52B—C52—H52C	108.2
C10—C3—C4	109.15 (17)	C60—C53—C52	109.65 (18)
C10—C3—C2	109.64 (16)	C60—C53—C54	109.59 (17)
C4—C3—C2	109.33 (17)	C52—C53—C54	109.72 (17)
C10—C3—H3B	109.6	C60—C53—H53B	109.3
C4—C3—H3B	109.6	C52—C53—H53B	109.3
C2—C3—H3B	109.6	C54—C53—H53B	109.3
C5—C4—C3	109.98 (17)	C55—C54—C53	109.22 (17)
C5—C4—H4B	109.7	C55—C54—H54B	109.8
C3—C4—H4B	109.7	C53—C54—H54B	109.8
C5—C4—H4C	109.7	C55—C54—H54C	109.8
C3—C4—H4C	109.7	C53—C54—H54C	109.8
H4B—C4—H4C	108.2	H54B—C54—H54C	108.3
C4—C5—C6	109.03 (18)	C54—C55—C56	109.45 (17)
C4—C5—C9	109.24 (17)	C54—C55—C59	108.79 (17)
C6—C5—C9	109.56 (17)	C56—C55—C59	109.54 (17)
C4—C5—H5A	109.7	C54—C55—H55A	109.7
C6—C5—H5A	109.7	C56—C55—H55A	109.7
C9—C5—H5A	109.7	C59—C55—H55A	109.7
C7—C6—C5	109.09 (17)	C57—C56—C55	109.45 (17)
C7—C6—H6A	109.9	C57—C56—H56A	109.8
C5—C6—H6A	109.9	C55—C56—H56A	109.8
C7—C6—H6B	109.9	C57—C56—H56B	109.8
C5—C6—H6B	109.9	C55—C56—H56B	109.8
H6A—C6—H6B	108.3	H56A—C56—H56B	108.2
C10—C7—C6	109.60 (17)	C60—C57—C56	109.76 (18)
C10—C7—C8	109.20 (17)	C60—C57—C58	108.50 (17)
C6—C7—C8	109.73 (17)	C56—C57—C58	109.97 (17)
C10—C7—H7A	109.4	C60—C57—H57A	109.5
C6—C7—H7A	109.4	C56—C57—H57A	109.5
C8—C7—H7A	109.4	C58—C57—H57A	109.5
C1—C8—C7	111.05 (16)	C51—C58—C57	110.60 (17)
C1—C8—H8A	109.4	C51—C58—H58A	109.5
C7—C8—H8A	109.4	C57—C58—H58A	109.5
C1—C8—H8B	109.4	C51—C58—H58B	109.5
C7—C8—H8B	109.4	C57—C58—H58B	109.5
H8A—C8—H8B	108.0	H58A—C58—H58B	108.1
C5—C9—C1	110.86 (17)	C55—C59—C51	111.33 (16)
C5—C9—H9A	109.5	C55—C59—H59A	109.4
C1—C9—H9A	109.5	C51—C59—H59A	109.4
C5—C9—H9B	109.5	C55—C59—H59B	109.4
C1—C9—H9B	109.5	C51—C59—H59B	109.4
H9A—C9—H9B	108.1	H59A—C59—H59B	108.0
C7—C10—C3	109.30 (17)	C57—C60—C53	109.58 (17)
C7—C10—H10A	109.8	C57—C60—H60A	109.8
C3—C10—H10A	109.8	C53—C60—H60A	109.8
C7—C10—H10B	109.8	C57—C60—H60B	109.8
C3—C10—H10B	109.8	C53—C60—H60B	109.8
H10A—C10—H10B	108.3	H60A—C60—H60B	108.2
C12—C11—C1	117.28 (16)	C62—C61—C51	116.11 (17)
C12—C11—H11A	108.0	C62—C61—H61A	108.3
C1—C11—H11A	108.0	C51—C61—H61A	108.3
C12—C11—H11B	108.0	C62—C61—H61B	108.3
C1—C11—H11B	108.0	C51—C61—H61B	108.3
H11A—C11—H11B	107.2	H61A—C61—H61B	107.4
C17—C12—C13	116.8 (2)	C67—C62—C63	117.53 (18)
C17—C12—C11	121.9 (2)	C67—C62—C61	121.10 (19)
C13—C12—C11	121.3 (2)	C63—C62—C61	121.38 (18)
C14—C13—C12	122.5 (2)	C64—C63—C62	121.09 (19)
C14—C13—H13A	118.8	C64—C63—H63A	119.5
C12—C13—H13A	118.8	C62—C63—H63A	119.5
C13—C14—C15	119.6 (2)	C63—C64—C65	120.62 (19)
C13—C14—H14A	120.2	C63—C64—H64A	119.7
C15—C14—H14A	120.2	C65—C64—H64A	119.7
C16—C15—C14	119.2 (2)	C64—C65—C66	119.31 (19)
C16—C15—N1	122.3 (2)	C64—C65—N51	117.14 (18)
C14—C15—N1	118.37 (19)	C66—C65—N51	123.53 (18)
C17—C16—C15	120.1 (2)	C67—C66—C65	118.95 (19)
C17—C16—H16A	120.0	C67—C66—H66A	120.5
C15—C16—H16A	120.0	C65—C66—H66A	120.5
C16—C17—C12	121.9 (2)	C62—C67—C66	122.47 (19)
C16—C17—H17A	119.0	C62—C67—H67A	118.8
C12—C17—H17A	119.0	C66—C67—H67A	118.8
N1—C18—N2	122.07 (19)	N51—C68—N52	121.44 (19)
N1—C18—C21	119.73 (19)	N51—C68—C71	120.41 (18)
N2—C18—C21	118.20 (19)	N52—C68—C71	118.15 (18)
N3—C19—N2	132.44 (19)	N53—C69—N52	131.12 (18)
N3—C19—Cl1	113.81 (16)	N53—C69—Cl51	114.79 (15)
N2—C19—Cl1	113.75 (16)	N52—C69—Cl51	114.09 (15)
N3—C20—N5	126.69 (19)	N53—C70—N55	126.88 (18)
N3—C20—C21	127.4 (2)	N53—C70—C71	126.86 (19)
N5—C20—C21	105.88 (18)	N55—C70—C71	106.25 (17)
C20—C21—N4	110.77 (18)	N54—C71—C70	110.81 (17)
C20—C21—C18	116.36 (19)	N54—C71—C68	132.71 (18)
N4—C21—C18	132.77 (19)	C70—C71—C68	116.44 (18)
N4—C22—N5	114.56 (18)	N54—C72—N55	114.83 (18)
N4—C22—H22A	122.7	N54—C72—H72A	122.6
N5—C22—H22A	122.7	N55—C72—H72A	122.6
N5—C23—C25	110.12 (17)	N55—C73—C74	110.12 (16)
N5—C23—C24	109.93 (18)	N55—C73—C75	109.84 (16)
C25—C23—C24	112.2 (2)	C74—C73—C75	112.51 (18)
N5—C23—H23A	108.1	N55—C73—H73A	108.1
C25—C23—H23A	108.1	C74—C73—H73A	108.1
C24—C23—H23A	108.1	C75—C73—H73A	108.1
C23—C24—H24A	109.5	C73—C74—H74A	109.5
C23—C24—H24B	109.5	C73—C74—H74B	109.5
H24A—C24—H24B	109.5	H74A—C74—H74B	109.5
C23—C24—H24C	109.5	C73—C74—H74C	109.5
H24A—C24—H24C	109.5	H74A—C74—H74C	109.5
H24B—C24—H24C	109.5	H74B—C74—H74C	109.5
C23—C25—H25A	109.5	C73—C75—H75A	109.5
C23—C25—H25B	109.5	C73—C75—H75B	109.5
H25A—C25—H25B	109.5	H75A—C75—H75B	109.5
C23—C25—H25C	109.5	C73—C75—H75C	109.5
H25A—C25—H25C	109.5	H75A—C75—H75C	109.5
H25B—C25—H25C	109.5	H75B—C75—H75C	109.5
			
C8—C1—C2—C3	58.5 (2)	C58—C51—C52—C53	58.5 (2)
C9—C1—C2—C3	−58.6 (2)	C59—C51—C52—C53	−59.0 (2)
C11—C1—C2—C3	177.92 (16)	C61—C51—C52—C53	178.21 (16)
C1—C2—C3—C10	−59.9 (2)	C51—C52—C53—C60	−59.7 (2)
C1—C2—C3—C4	59.7 (2)	C51—C52—C53—C54	60.7 (2)
C10—C3—C4—C5	60.3 (2)	C60—C53—C54—C55	59.9 (2)
C2—C3—C4—C5	−59.6 (2)	C52—C53—C54—C55	−60.6 (2)
C3—C4—C5—C6	−60.2 (2)	C53—C54—C55—C56	−60.1 (2)
C3—C4—C5—C9	59.5 (2)	C53—C54—C55—C59	59.5 (2)
C4—C5—C6—C7	60.0 (2)	C54—C55—C56—C57	60.2 (2)
C9—C5—C6—C7	−59.5 (2)	C59—C55—C56—C57	−59.0 (2)
C5—C6—C7—C10	−60.5 (2)	C55—C56—C57—C60	−59.9 (2)
C5—C6—C7—C8	59.4 (2)	C55—C56—C57—C58	59.4 (2)
C2—C1—C8—C7	−58.8 (2)	C52—C51—C58—C57	−59.2 (2)
C9—C1—C8—C7	58.6 (2)	C59—C51—C58—C57	58.0 (2)
C11—C1—C8—C7	179.78 (17)	C61—C51—C58—C57	179.16 (17)
C10—C7—C8—C1	60.1 (2)	C60—C57—C58—C51	60.4 (2)
C6—C7—C8—C1	−60.0 (2)	C56—C57—C58—C51	−59.7 (2)
C4—C5—C9—C1	−59.2 (2)	C54—C55—C59—C51	−60.2 (2)
C6—C5—C9—C1	60.1 (2)	C56—C55—C59—C51	59.4 (2)
C2—C1—C9—C5	58.4 (2)	C52—C51—C59—C55	59.4 (2)
C8—C1—C9—C5	−58.7 (2)	C58—C51—C59—C55	−58.4 (2)
C11—C1—C9—C5	−178.01 (17)	C61—C51—C59—C55	−177.72 (17)
C6—C7—C10—C3	60.6 (2)	C56—C57—C60—C53	59.7 (2)
C8—C7—C10—C3	−59.6 (2)	C58—C57—C60—C53	−60.5 (2)
C4—C3—C10—C7	−60.1 (2)	C52—C53—C60—C57	60.8 (2)
C2—C3—C10—C7	59.7 (2)	C54—C53—C60—C57	−59.7 (2)
C2—C1—C11—C12	59.7 (2)	C52—C51—C61—C62	57.5 (2)
C8—C1—C11—C12	178.91 (18)	C58—C51—C61—C62	177.43 (17)
C9—C1—C11—C12	−62.1 (2)	C59—C51—C61—C62	−63.3 (2)
C1—C11—C12—C17	−94.6 (2)	C51—C61—C62—C67	−98.4 (2)
C1—C11—C12—C13	87.5 (2)	C51—C61—C62—C63	81.8 (2)
C17—C12—C13—C14	0.4 (3)	C67—C62—C63—C64	1.5 (3)
C11—C12—C13—C14	178.31 (18)	C61—C62—C63—C64	−178.71 (18)
C12—C13—C14—C15	−0.1 (3)	C62—C63—C64—C65	0.1 (3)
C13—C14—C15—C16	−0.2 (3)	C63—C64—C65—C66	−1.2 (3)
C13—C14—C15—N1	176.24 (17)	C63—C64—C65—N51	176.95 (18)
C18—N1—C15—C16	−37.7 (3)	C68—N51—C65—C64	160.18 (19)
C18—N1—C15—C14	146.1 (2)	C68—N51—C65—C66	−21.7 (3)
C14—C15—C16—C17	0.1 (3)	C64—C65—C66—C67	0.8 (3)
N1—C15—C16—C17	−176.15 (17)	N51—C65—C66—C67	−177.28 (18)
C15—C16—C17—C12	0.2 (3)	C63—C62—C67—C66	−2.0 (3)
C13—C12—C17—C16	−0.4 (3)	C61—C62—C67—C66	178.24 (19)
C11—C12—C17—C16	−178.37 (18)	C65—C66—C67—C62	0.9 (3)
C15—N1—C18—N2	−5.6 (3)	C65—N51—C68—N52	−6.8 (3)
C15—N1—C18—C21	174.88 (19)	C65—N51—C68—C71	173.91 (19)
C19—N2—C18—N1	179.85 (18)	C69—N52—C68—N51	178.00 (18)
C19—N2—C18—C21	−0.6 (3)	C69—N52—C68—C71	−2.7 (3)
C20—N3—C19—N2	2.2 (3)	C70—N53—C69—N52	5.2 (3)
C20—N3—C19—Cl1	−177.30 (14)	C70—N53—C69—Cl51	−175.13 (14)
C18—N2—C19—N3	−2.6 (3)	C68—N52—C69—N53	−3.6 (3)
C18—N2—C19—Cl1	177.00 (14)	C68—N52—C69—Cl51	176.80 (14)
C19—N3—C20—N5	−178.37 (19)	C69—N53—C70—N55	177.51 (19)
C19—N3—C20—C21	1.2 (3)	C69—N53—C70—C71	−0.9 (3)
C22—N5—C20—N3	−179.8 (2)	C72—N55—C70—N53	−177.88 (19)
C23—N5—C20—N3	1.1 (3)	C73—N55—C70—N53	4.3 (3)
C22—N5—C20—C21	0.5 (2)	C72—N55—C70—C71	0.8 (2)
C23—N5—C20—C21	−178.52 (18)	C73—N55—C70—C71	−176.98 (17)
N3—C20—C21—N4	179.46 (19)	C72—N54—C71—C70	0.5 (2)
N5—C20—C21—N4	−0.9 (2)	C72—N54—C71—C68	−176.9 (2)
N3—C20—C21—C18	−3.8 (3)	N53—C70—C71—N54	177.86 (18)
N5—C20—C21—C18	175.82 (17)	N55—C70—C71—N54	−0.8 (2)
C22—N4—C21—C20	0.9 (2)	N53—C70—C71—C68	−4.3 (3)
C22—N4—C21—C18	−175.1 (2)	N55—C70—C71—C68	176.99 (17)
N1—C18—C21—C20	−177.17 (19)	N51—C68—C71—N54	2.5 (3)
N2—C18—C21—C20	3.3 (3)	N52—C68—C71—N54	−176.80 (19)
N1—C18—C21—N4	−1.4 (3)	N51—C68—C71—C70	−174.68 (17)
N2—C18—C21—N4	179.1 (2)	N52—C68—C71—C70	6.0 (3)
C21—N4—C22—N5	−0.6 (2)	C71—N54—C72—N55	0.1 (2)
C20—N5—C22—N4	0.1 (2)	C70—N55—C72—N54	−0.6 (2)
C23—N5—C22—N4	179.07 (19)	C73—N55—C72—N54	177.10 (18)
C22—N5—C23—C25	−59.4 (3)	C72—N55—C73—C74	26.2 (3)
C20—N5—C23—C25	119.4 (2)	C70—N55—C73—C74	−156.61 (18)
C22—N5—C23—C24	64.7 (3)	C72—N55—C73—C75	−98.3 (2)
C20—N5—C23—C24	−116.5 (2)	C70—N55—C73—C75	78.9 (2)

### Hydrogen-bond geometry (Å, °)

**Table:**

*D*—H···*A*	*D*—H	H···*A*	*D*···*A*	*D*—H···*A*
N1—H1A···N54	0.88	2.14	2.940 (2)	152
N51—H51A···N4	0.88	2.27	3.026 (2)	144

## References

[bb1] Farrugia, L. J. (1997). *J. Appl. Cryst.* **30**, 565.

[bb2] Fiorini, M. T. & Abell, C. (1998). *Tetrahedron Lett.* **39**, 1827–1830.

[bb3] Hardcastle, I. R., Golding, B. T. & Griffin, R. J. (2002). *Annu. Rev. Pharmacol. Toxicol.* **42**, 325–348. 10.1146/annurev.pharmtox.42.090601.125940 11807175

[bb4] Oxford Diffraction (2006). *CrysAlis CCD* and *CrysAlis RED* Oxford Diffraction Ltd, Abingdon, England.

[bb5] Rouchal, M., Nečas, M., de Carvalho, F. P. & Vícha, R. (2009*a*). *Acta Cryst.* E**65**, o298–o299. 10.1107/S160053680900052X PMC2968337 21581908

[bb6] Rouchal, M., Nečas, M. & Vícha, R. (2009*b*). *Acta Cryst.* E**65**, o1268. 10.1107/S1600536809016596 PMC2969555 21583132

[bb7] Sheldrick, G. M. (2008). *Acta Cryst.* A**64**, 112–122. 10.1107/S0108767307043930 18156677

[bb8] Trávníček, Z. & Popa, I. (2007*a*). *Acta Cryst.* E**63**, o629–o631.

[bb9] Trávníček, Z. & Popa, I. (2007*b*). *Acta Cryst.* E**63**, o728–o730.

[bb10] Trávníček, Z. & Zatloukal, M. (2004). *Acta Cryst.* E**60**, o924–o926.

[bb11] Villhauer, E. B., Brinkman, J. A., Naderi, G. B., Burkey, B. F., Dunning, B. E., Prasad, K., Mangold, B. L., Russell, M. E. & Hughes, T. E. (2003). *J. Med. Chem.* **46**, 2774–2789. 10.1021/jm030091l 12801240

